# The roles of transition metals in the physiology and pathogenesis of *Streptococcus pneumoniae*

**DOI:** 10.3389/fcimb.2013.00092

**Published:** 2013-12-04

**Authors:** Erin S. Honsa, Michael D. L. Johnson, Jason W. Rosch

**Affiliations:** Department of Infectious Diseases, St. Jude Children's Research HospitalMemphis, TN, USA

**Keywords:** *Streptococcus pneumoniae*, pathogenesis, metal transport, virulence factors, infection

## Abstract

For bacterial pathogens whose sole environmental reservoir is the human host, the acquisition of essential nutrients, particularly transition metals, is a critical aspect of survival due to tight sequestration and limitation strategies deployed to curtail pathogen outgrowth. As such, these bacteria have developed diverse, specialized acquisition mechanisms to obtain these metals from the niches of the body in which they reside. To oppose the spread of infection, the human host has evolved multiple mechanisms to counter bacterial invasion, including sequestering essential metals away from bacteria and exposing bacteria to lethal concentrations of metals. Hence, to maintain homeostasis within the host, pathogens must be able to acquire necessary metals from host proteins and to export such metals when concentrations become detrimental. Furthermore, this acquisition and efflux equilibrium must occur in a tissue-specific manner because the concentration of metals varies greatly within the various microenvironments of the human body. In this review, we examine the functional roles of the metal import and export systems of the Gram-positive pathogen *Streptococcus pneumoniae* in both signaling and pathogenesis.

## Metals and infection

*Streptococcus pneumoniae* can cause a variety of infections, including meningitis, otitis media, bacteremia, and pneumonia—the infection causing the most deaths worldwide from this pathogen (Wardlaw et al., 2006). During these various forms of bacterial infection, *S. pneumoniae* must acquire all necessary nutrients for survival and replication from within the host. Transition metals are an important subset of nutrients because they are required as cofactors and structural components of many proteins and play vital roles in metabolism and cellular defenses (Andreini et al., 2008). The bioavailability of metals in various host sites of pneumococcal colonization and infection vary significantly, which is reflected by the contribution to virulence of different pneumococcal metal import and export systems in these various host niches.

Although the efficient acquisition of metals is important, over-accumulation of intracellular metals can have deleterious effects on multiple cellular pathways, including antioxidant defense and central metabolic pathways. As such, bacteria have evolved highly efficient efflux mechanisms and precise regulatory systems to ensure appropriate modulation of intracellular metal accumulation. In addition to the concentration of a particular metal, the relative concentration of a particular metal in relation to that of other metals is a vital aspect of bacterial physiology because these metals can compete for intracellular binding sites within proteins (Dudev and Lim, 2008).

**Figure 1:**
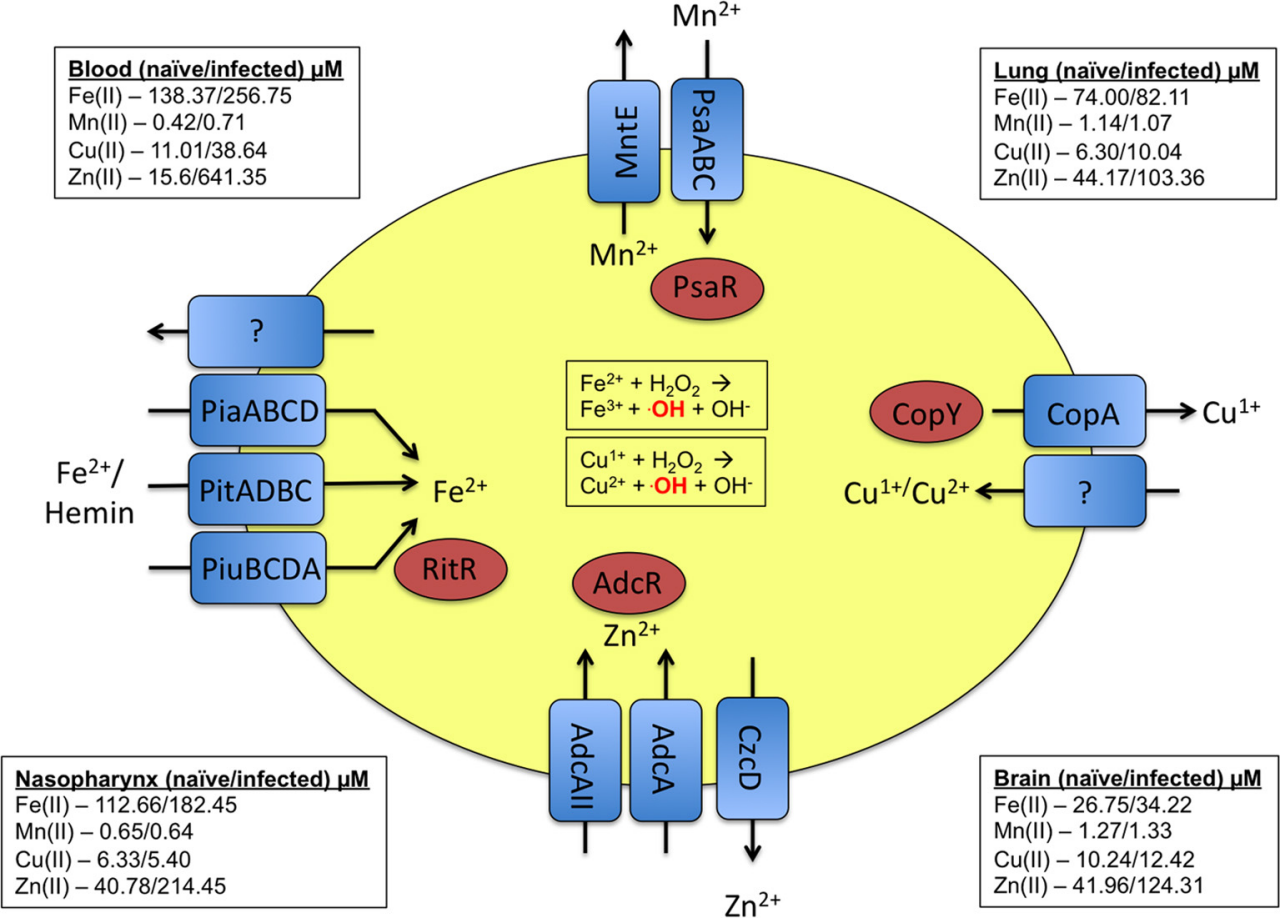
**Summary of the metal uptake and efflux systems of pneumococcus.** Transporters and their respective substrates are indicated in blue. The regulators that respond to the respective metals are indicated in red. As a point of reference, the levels of the discussed metals at various host sites in both naïve and infected animals are provided in the corners of the diagram (McDevitt et al., 2011).

Baseline levels of metals vary greatly between various sites in the human body. As a reference, we have provided published concentrations of these metals in various body sites (McDevitt et al., 2011), as well as the pneumococcal transporters associated with these transition metals (Figure 1). In response to infection and inflammation, the bioavailability of metals can be rapidly altered, with the host sequestering nutrients from the bacterium to limit bacterial growth (Corbin et al., 2008; Weinberg, 2009; White et al., 2009). For example, the calprotectin protein chelates zinc and manganese during infection, rendering them unavailable to the pathogenic bacterium (Kehl-Fie and Skaar, 2010). In response to pneumococcal infection, the levels of metals in host tissues can vary dramatically. For example, zinc in the blood increases more than 10-fold during infection (McDevitt et al., 2011). Another example of the host modulating metal concentrations occurs within the phagolysosome of immune cells. Here, the innate immune cell actively pumps out necessary metals such as manganese and iron and pumps in toxic metals such as copper and zinc to eliminate the pathogen (Jabado et al., 2000; Forbes and Gros, 2003; White et al., 2009; Botella et al., 2011). This arms race for metals can determine whether or not an infection would be successful. In this review, we examine the role of the transition metals manganese, iron, copper, and zinc in *S. pneumoniae* physiology and pathogenesis.

## Manganese

Although metals are toxic to bacteria, which require export systems for sustained viability, metal ion homeostasis must be maintained because metals are essential for bacterial viability and survival. For *S. pneumoniae*, one such metal that is critical for sustained colonization and invasive disease is manganese (Mn). Mn^2+^ is found in various concentrations within the human host, depending on the body site, and is an essential cofactor for many pneumococcal proteins. In this section, we will discuss the specific roles that this divalent metal plays in *S. pneumoniae* growth and virulence, as well as the antioxidant properties of Mn^2+^. Table 1 lists all Mn^2+^-related proteins and genes of *S. pneumoniae* that will be discussed.

**Table 1:** **Summary of *Streptococcus pneumoniae* genes and their products involved in transition metal import/export, and metal-dependent proteins**.

**Operon**	**Gene**	**SP number (TIGR4)**	**Protein product**	**Role in virulence (mutant)**	**Reference/s**
*psaABC* (Manganese import)	*psaA*	SP_1650	Manganese ABC transporter substrate-binding lipoprotein	Highly attenuated (otitis media, respiratory infection, systemic invasion).	Berry and Paton, 1996; Marra et al., 2002
	*psaB*	SP_1648	Manganese ABC transporter ATP-binding protein	Absolute requirement for Mn^2+^ in growth media.	Berry and Paton, 1996; Johnston et al., 2006
	*psaC*	SP_1649	Manganese ABC transporter permease	Absolute requirement for Mn^2+^ in growth media.	Berry and Paton, 1996; Johnston et al., 2006
	*psaR*	SP_1638	Manganese-dependent regulator	Essential for murine systemic infection. Dispensable for nasopharyngeal colonization.	Johnston et al., 2006
	*sodA*	SP_0766	Manganese-dependent superoxide dismutase	Dispensible for systemic infection. Essential for initial lung infection and invasion into the bloodstream.	Yesilkaya et al., 2000
	*mntE*	SP_1552	Mn^2+^ efflux membrane protein	Important for colonization of nasopharynx and establishing a systemic infection.	Rosch et al., 2009
*piuBCDA* (Iron import)	*piuA*	SP_1872	Iron lipoprotein receptor	No effect on pneumonia or systemic modes of infection. Double mutant with the pia system demonstrated delayed time to death in systemic infection.	Brown et al., 2001a, 2002
	*piuB*	SP_1869	Transmembrane permease		
	*piuC*	SP_1870	Transmembrane permease		
	*piuD*	SP_1871	ATPase component		
*piaABCD* (Iron import)	*piaA*	SP_1032	Iron lipoprotein receptor	No effect on pneumonia or systemic modes of infection. Double mutant with the piu system demonstrated delayed time to death in systemic infection.	Brown et al., 2001a, 2002
	*piaB*	SP_1033	Transmembrane permease		
	*piaC*	SP_1034	Transmembrane permease		
	*piaD*	SP_1035	ATPase component		
*pitADBC* (Iron import)	*pitA*	SP_0240	Iron lipoprotein receptor	Deletion of operon had no effect on pneumonia model of infection. Slight role in systemic infection. Additive affect when knocked out with piu and pia systems.	Brown et al., 2002
	*pitB*	SP_0242	Transmembrane permease		
	*pitC*	SP0243	Transmembrane permease		
	*pitD*	SP_0241	ATPase component		
	*ritR*	SP_0376	Repressor of iron transport	Required for murine pneumonia.	Throup et al., 2000; Ulijasz et al., 2004
*copY/cupA/copA* (Copper export)	*copY*	SP_0727	*cop operon regulator*	N/A	Shafeeq et al., 2011b
	*cupA*	SP_0728	Copper transport protein and potential cupredoxin	Slightly More Sensitive Than Wild Type to copper in growth media	Shafeeq et al., 2011b
	*copA*	SP_0729	P-type ATPase copper exporter	Slightly More Sensitive Than Wild Type to copper in growth media; reduced virulence in colonization of nasopharynx	Shafeeq et al., 2011b; Fu et al., 2013
*adcAAIIBCR* (Zinc import)	*adcA*	SP_2169	Zinc Importer (Redundant with AdcAII)	No effect	McDevitt et al., 2011
	*adcAII*	SP_1002	Zinc Importer (Redundant with AdcA)	No effect	McDevitt et al., 2011
	*adcB*	SP_2170	Transmembrane permease	Significantly more sensitive than wild type	McDevitt et al., 2011
	*adcC*	SP_2171	ATPase	No effect	Hava and Camilli, 2002; van Opijnen and Camilli, 2012; Kimaro Mlacha et al., 2013
	*adcR*	SP_2172	Import Machinery Transcriptional Reguator	N/A	N/A
	*czcD*	1857	Zinc efflux system	No effect	Hava and Camilli, 2002; van Opijnen and Camilli, 2012; Kimaro Mlacha et al., 2013;

N/A, not available.

### Manganese importer: PsaBCA

The pneumococcal Mn^2+^-importer is the PsaBCA ATP-binding cassette (ABC) transporter, which acquires and pumps Mn^2+^ ions from the extracellular environment (i.e., mammalian host) into the cytosol. PsaBCA belongs to the Cluster A-I substrate binding protein transporters, which can transport Mn^2+^, Fe^2+^, and Zn^2+^ (Figure 1). However, deletion of the transmembrane and ATP-binding components (PsaC and PsaB, respectively) results in an absolute requirement for Mn^2+^ in the growth medium (Novak et al., 1998; Johnston et al., 2006; Berntsson et al., 2010), suggesting that Mn^2+^ is the transporter's preferred substrate. Furthermore, cells lacking the PsaA component are deficient in growth in defined media lacking Mn^2+^ (Dintilhac et al., 1997; Novak et al., 1998).

The PsaA component was the first member of the Cluster A-I family substrate binding transportation proteins to be crystallized, with structures showing a Zn^2+^ ion within the metal-coordination site. However, free Zn^2+^ is unable to downregulate PsaBCA expression, suggesting that Zn^2+^ is not the natural ligand of PsaA (Kloosterman et al., 2008). Two published studies have shown that PsaA binds to Zn^2+^ with a higher affinity than it does to Mn^2+^ and that PsaA-Zn^2+^ binding is responsible for an ~40% reduction in Mn^2+^ accumulation in the cytosol (Jacobsen et al., 2011; McDevitt et al., 2011). Zn^2+^ binding to PsaA, therefore, partially inhibits Mn^2+^ binding and subsequent import. This phenomenon of inhibiting nutrient acquisition by the bacterium may partially explain why extracellular Zn^2+^ is toxic to *S. pneumoniae*, as will be discussed later.

PsaA's mechanism of Mn^2+^ import has recently been determined through a series of elegant structural and biochemical experiments (Counago et al., 2013). The metal-coordinating residues of PsaA were shown to interact with both Mn^2+^ and Zn^2+^, similar to previously published observations, with the subsequent release and intracellular import of Mn^2+^ found to be facilitated by the sub-optimal coordination of Mn^2+^ in the metal-binding site. In contrast, optimal coordination of zinc resulted in the locking of the conformation of PsaA in a manner whereby the zinc ion was not released. This situation highlights the importance of ligand specificity and biological function, as many such import machineries must discriminate between metals of varying reactivity and abundance in the host.

### Manganese-dependent gene regulation

The *psaBCA* operon repressor PsaR negatively regulates the Psa import machinery. The recently elucidated crystal structure of the *S. pneumoniae* PsaR forms a stable homodimer and contains two metal-binding sites (Lisher et al., 2013). Site one coordinates Zn^2+^ with a *K*_D_ ≥ 10^13^ M^−1^. Site two mediates metal selectivity and DNA activation of PsaR. Although Zn^2+^ bound to this site with a higher affinity than did Mn^2+^, Mn^2+^ was essential for the activation of DNA binding by PsaR. This requirement for Mn^2+^ is due to the higher allosteric coupling free energy released when Mn^2+^ binds to site two than when Zn^2+^ binds. Altogether, this study elucidated the first PsaR crystal structure and showed that PsaR requires Mn^2+^ to be bound within site two for DNA binding activity, and most likely *psaBCA* gene repression.

Mn^2+^ also plays an important role in the ability of the pneumococcus to regulate its response to stressors (Balogun et al., 2010). Global transcriptomic and proteomic analyses of WT and Δ*psaA* pneumococci, grown under high- or low-Mn^2+^, revealed the overall effect of Mn^2+^ on various biological processes of the pneumococcus. In the absence of supplemental Mn^2+^, the *psaBCA* gene expression was increased, consistent with the Mn-dependent binding of PsaR (repressor) to the promoter of the *psa* operon, when excess Mn2+ was present in the bacterial cytosol (Johnston et al., 2006). Additional genes were also controlled by the PsaR regulator, including the virulence factors *prtA* (*sp0641*) and *pcpA* (*sp2136*), which encode a cell wall-associated serine protease and CbpA, respectively (Hoskins et al., 2001).

Mn^2+^ has also been implicated in the development of natural competence in the pneumococcus. In fact, the Δ*psaA* strain absolutely requires Mn^2+^ supplementation in the growth media for competence and transformation (Dintilhac et al., 1997). Transcriptome analysis results provided the first real evidence that PsaA was needed to induce competence. Because PsaA is also required for Mn^2+^ import, this finding suggested that a certain level of Mn^2+^ must be present in the cytosol to trigger competence. Indeed, the competence transcriptional regulator *comX1*, the competence operon, and the competence-stimulating peptide were all downregulated in strains lacking *psaA*.

### The role of manganese in protection against oxidative stress

When the expression of the ligand-binding lipoprotein *psaA* was knocked out, the resultant Δ*psaA* pneumococcus was hypersensitive to oxidative stress and had lowered ability to neutralize the reactive oxygen species (ROS) superoxide anions (Tseng et al., 2002). For *S. pneumoniae*, ROS accumulation is a particularly volatile situation because this bacterium does not express a catalase, an enzyme essential for breaking down and neutralizing H_2_O_2_ (Hoskins et al., 2001). Furthermore, during the normal function of the pyruvate oxidase SpxB, which is required for the decarboxylation of pyruvate to acetyl phosphate and CO_2_, high levels of H_2_O_2_ are produced as a byproduct (Spellerberg et al., 1996). In fact, when SpxB is absent from a mutant strain of pneumococcus, the relative levels of cytosolic H_2_O_2_ drop 99%, suggesting that SpxB is the major H_2_O_2_-producing protein. Therefore, the pneumococcus and many other bacteria have evolved systems to break down ROS molecules to prevent oxidative stress. Because the Δ*psaA* mutant is unable to acquire Mn^2+^, it is proposed that the loss of the antioxidant property of Mn^2+^ is responsible of the accumulation of ROS, resulting in oxidative damage and cell death (Spellerberg et al., 1996). Although Mn^2+^ is a cofactor for many proteins, the Mn-bicarbonate complex can also reduce ROS molecules directly, without the need for enzymes (Daly et al., 2004). However, this Mn-dependent ROS neutralization activity has not yet been found in the pneumococcus.

The superoxide anion (O^−^_2_) is a major source of oxidative stress within the pneumococcus. Sod metalloenzymes neutralize superoxide anions by converting them to molecular water and H_2_O_2_, and are a major cellular defense against oxidative stress (McCord and Fridovich, 1969; Hassan, 1989). Although the production of H_2_O_2_ itself may be deleterious to the pneumococcus, previous results suggest that the Mn-dependent superoxide dismutase SodA is responsible for only 1% of pneumococcal H_2_O_2_ production. Furthermore, as we have discussed, SpxB was responsible for the remaining 99% (Spellerberg et al., 1996). Therefore, although the mechanism of H_2_O_2_ neutralization by the pneumococcus remains unknown, the role of SodA is highly critical for the survival and virulence of the pneumococcus when grown in a high oxidative stress environment.

Yesilkaya et al. reported the first detailed information on the pneumococcal Mn-dependent SodA, its role in protecting against oxidative stress, and its possible role in virulence (Yesilkaya et al., 2000). They showed that the pneumococcus produces two Sod proteins: a Mn-dependent Sod (SodA) and an iron-dependent Sod that is inhibited by H_2_O_2_. The pneumococcal genome clearly contained *sodA*, but no other discernible superoxide dismutase homologs have been identified to date. It should be noted that the presence of an iron-dependent SodA has not been addressed since the original study. Deleting *sodA* impairs pneumococcal growth under aerobic conditions, most likely due to the loss of the Mn-driven neutralization of O^−^_2_ produced during growth in a high molecular oxygen environment (Yesilkaya et al., 2000). Furthermore, the mutation is lethal under anaerobic conditions. The authors suggest that this result may be due to the loss of superoxide scavenging function during anaerobic growth in this highly limiting growth medium and that the cells are highly susceptible to death by oxidative stress without the Mn-dependent SodA.

### Manganese efflux

As with all biological systems, pneumococci must achieve equilibrium between the amount of nutrient acquired and the amount of nutrient required. In the case of Mn^2+^, which serves a positive role in the growth and virulence of the pneumococcus, there may be negative side effects to its accumulation within the cytosol if levels exceed those needed. To help control this tight regulation, the pneumococcus encodes a Mn^2+^-efflux protein MntE, which selectively removes excess Mn^2+^ from the cytosol to maintain metal homeostasis (Figure 1) (Rosch et al., 2009). MntE was originally predicted to be a CDF inorganic cation transporter, however, its substrate was unknown (Tettelin et al., 2001). Subsequent analysis of a Δ*mntE* mutant showed its enhanced sensitivity to high levels of Mn^2+^ in the growth medium, attributed to the inability of the mutant to export internal Mn^2+^ ions (Rosch et al., 2009). Also, the levels of intracellular Mn^2+^ in the mutant were significantly higher than those of the parental strain. These data indicate that MntE functions as a manganese efflux transporter in the pneumococcus.

### Manganese homeostasis and virulence

Based on the importance of manganese to the pneumococcus, it was not unexpected that the Δ*psaA* mutant was highly attenuated for multiple modes of pneumococcal infection, including otitis media, respiratory infection, and systemic invasion (Berry and Paton, 1996; Marra et al., 2002). One of the critical steps in pneumococcal pathogenesis is the initial adherence to and invasion of host cells, and a Δ*psaA* strain of pneumococcus has decreased adherence to mammalian cells (Berry and Paton, 1996). Although it was originally hypothesized that PsaA was a surface-exposed adhesin, subsequent studies showed that PsaA plays a predominantly regulatory role in adherence, attributed to the important cellular role of the Mn-dependent repressor PsaR. Furthermore, perturbing manganese import affected the levels of the structural adhesin CbpA, an important virulence determinant for the pneumococcus, on the bacterial surface (Novak et al., 1998). It was, therefore, proposed that Mn-import by PsaBCA and the Mn-homeostasis role of PsaR are ultimately responsible for patterns of CbpA expression and pneumococcal adherence (Paton, 1998). Subsequent work showed that although PsaR is responsible for the negative expression of the Mn-transporter PsaBCA and several other surface proteins, PsaR inactivation does not alter pneumococcal adherence to cultured nasopharyngeal cells (Johnston et al., 2006). Also, though PsaR-mediated repression is essential for the pneumococcus to establish a systemic infection, Mn-dependent signaling through PsaR is not required for nasopharyngeal colonization (Johnston et al., 2006). This discrepancy may be due to the differences between the Mn levels of the lungs and those of the nasopharynx, which are shown in Figure 1. Regardless, these findings highlight the importance of Mn^2+^ acquisition during pneumococcal infection.

The results of initial studies of intranasal models of infection suggested that SodA may also have a role in the initial colonization of the lung tissue and subsequent invasion into the bloodstream (Yesilkaya et al., 2000). To confirm this hypothesis, animals were intravenously infected with WT and *ΔsodA* pneumococcal strains. No differences in median survival times were observed, suggesting that SodA is dispensable during a systemic pneumococcal infection (Yesilkaya et al., 2000). These findings may suggest that the microenvironment of the lungs, being rich in molecular oxygen, is the site of maximal SodA activity, protecting against the high oxidative stress levels. The delay in infection as the bacteria traverse the oxygen-rich alveoli into the more micro-aerophilic bloodstream suggests the ability of Mn-SodA to neutralize cytosolic oxidative stress is a determinant of pneumococcal pathogenesis and that is dispensable once the bacteria reach the bloodstream. This finding highlights the importance of understanding the niche-specific contribution of virulence genes to pathogenesis.

Mn^2+^ accumulation in the cytosol increases *S. pneumoniae* resistance to oxidative stress (Rosch et al., 2009). This finding was expected because Mn^2+^ is an antioxidant and is the cofactor for SodA, which eliminates ROS from the cytosol. The high accumulation of Mn^2+^ in the *mntE* mutant can, however, be detrimental: increased internal concentrations of Mn^2+^ dysregulate the transcriptional profile of the pneumococcus, altering multiple cellular pathways (Rosch et al., 2009). Although the results of *in vitro* studies demonstrated that the *ΔmntE* pneumococcus is viable even under higher levels of oxidative stress, the results of *in vivo* pathogenicity studies indicate a fitness tradeoff for these benefits. The Δ*mntE* mutant has a significantly reduced ability to colonize the nasopharynx and establish systemic infection (Rosch et al., 2009). Therefore, communication at a transcriptional level in response to internal and external Mn^2+^ levels is of critical importance during disease progression.

### Targeting manganese homeostasis for vaccines

Because of both its surface location and important roles in virulence, PsaA has attracted considerable interest as a potential vaccine antigen for the pneumococcus. The current polyvalent pneumococcal vaccine is based on the polysaccharide capsule, of which there are more than 90 serotypes (Robbins and Schneerson, 1983; Robbins et al., 1983). Since the vaccine only protects against 23 of the most common serotypes, non-vaccine-type pneumococci have emerged that are capable of causing pneumonia and invasive diseases. Therefore, one major goal in the pneumococcal field is to develop a protein-based vaccine that targets one or more surface-exposed pneumococcal proteins. Candidate proteins must be present in all serotypes and elicit a strong serotype-independent immune response. Immunization of mice with PsaA conferred protection against invasive pneumococcal disease in a serotype-independent manner (Talkington et al., 1996). Inclusion of PsaA in multicomponent protein-based vaccines has also been efficacious for protection against both colonization and invasive disease (Briles et al., 2000; Ogunniyi et al., 2000). Hence, inclusion of such surface-exposed, metal-acquisition proteins may play an important role in the development of the next generation of pneumococcal vaccines.

## Iron

In the 1990s and early 2000s, substantial information was available about how Gram-negative pathogens acquired iron from their hosts (Crosa et al., 2004). Two main systems had been discovered: (1) small molecular siderophores that are synthesized and secreted by the bacteria to sequester free iron atoms or iron from host proteins such as transferrin; and (2) bacteria expressed surface proteins that bound hemin and imported the porphyrin into the cytosol for iron utilization. However, it remained unclear how Gram-positive organisms acquired iron through the thick cell wall and a single membrane.

### Host sources of iron used by pneumococci

In 1993, Tai and others began to uncover how the pneumococcus acquires iron from its host during systemic bacteremia (Tai et al., 1993). Free iron levels in the blood are ~10^−18^ M^−1^ in the mammalian host, with most iron sequestered within the tetrapyrrole ring of heme, which is bound to hemoglobin (Hb) in erythrocytes (De Domenico et al., 2008; Heinemann et al., 2008). Initial analysis focused on determining whether pneumococcal culture supernatants contain siderophores as do those from multiple Gram-negative and Gram-positive pathogens, including *Escherichia coli*, *Yersinia pestis*, *Haemophilus influenzae*, *Bacillus anthracis*, and *Staphylococcus aureus* (Crosa et al., 2004; Honsa and Maresso, 2011; Haley and Skaar, 2012). However, no siderophores were present in the supernatants of pneumococcal cultures grown in low-iron media, suggesting that the pneumococcus does not produce iron-scavenging siderophores for iron acquisition (Tai et al., 1993).

The possibility existed that the pneumococcus could acquire iron via hemin sources or other iron-atom acquisition systems such as transferrin-iron capture, as seen in *Neisseria meningitidis* and similar pathogens (Gray-Owen and Schryvers, 1996; Pintor et al., 1998). This hypothesis was tested by using pneumococci grown in EDTA-treated media whereby growth was inhibited due to low iron levels. Supplementation with iron-loaded (holo) transferrin or lactoferrin did not restore growth, suggesting that while the pneumococcus is a mucosal pathogen, it does not use these two host iron sources (Tai et al., 1993). However, when cells were grown in hemin or Hb, bacterial growth recovered to WT levels, providing the first evidence that the pneumococcus may exploit host hemin stores for iron during growth in the bloodstream. Furthermore, this was the first evidence that any Gram-positive pathogen could use hemin as an iron source.

Tai et al. continued to study possible hemin-binding and acquisition functions of the pneumococcus (Tai et al., 1997). They used a non-encapsulated strain of pneumococcus to detect heme-binding activity by cell wall proteins. As increasing concentrations of hemin were incubated with live pneumococci, the absorbance at 405 nm, a well-defined method to detect hemin in a sample (Berry and Trumpower, 1987), increased by nearly a 1:1 ratio with ~70% of hemin bound to the pneumococcal surface. Furthermore, pretreating cells at 75°C did not inhibit hemin binding, suggesting that hemin binding does not require metabolically active cells. Although this heating may destroy proteins that could be responsible for hemin binding, treating pneumococci with proteinase K also did not inhibit hemin association with the bacterial cell surface. In an attempt to identify the polypeptides responsible for hemin binding, batch affinity chromatography was performed, with hemin-coated agarose beads used to capture potential hemin-binding surface proteins (Tai et al., 1997). Several protein bands were seen, with the dominant species being 43 kDa, and adding free hemin to the cell lysate before column filtration eliminated the elution of the 43-kDa band. This 43-kDa protein was found in all pneumococcal serotypes tested and was predicted to be cell surface–exposed via fractionation results; hence, it was the most-promising candidate for a hemin-binding factor at the pneumococcal surface. Recently, researchers preliminarily identified two pneumococcal membrane proteins that bound hemin and Hb and were essential for viability (Romero-Espejel et al., 2013). As such these candidates show promise for the future understanding of how the pneumococcus acquires these complexes.

Elucidation of the details of iron transport can sometimes be confounded by the biochemistry of hemin and hemoglobin. One such difficulty can be non-specific binding of hemin to the bacterial cell surface, which can occur due to the hydrophobic nature of hemin. Another potential issue is that Hb can begin to dissociate during prolonged incubation: that is, heme will be oxidized to produce hemin and will dissociate from Hb subunits (Hargrove et al., 1997). Also, in some reported instances, hemin-binding proteins transiently bind porphyrin, and this event can be followed by a rapid dissociation of apo-hemin from the protein (Tsuruga and Shikama, 1997). As such, care must be taken to address these factors when investigating hemin and Hb utilization.

### The iron import machinery of pneumococcus

The ability to scavenge free iron atoms and the protein complexes responsible for this activity had not been discovered in the pneumococcus until two possible iron-import machineries were identified (Brown et al., 2001a). In this study, it was hypothesized that possible virulence determinants, which include iron importers, could be found in pathogenicity-associated islands. A previously performed signature-tagged mutagenesis screen used insertion-duplication mutants that were analyzed for their ability to survive and replicate in murine models of pneumonia and bacteremia. One gene, *smtA*, was attenuated for virulence, and was annotated as a Fe^3+^/-dicitrate ABC transporter (Figure 1) (Lau et al., 2001). This gene was predicted to encode a transmembrane permease and was initially termed the *pit1BCDA* operon (pneumococcal iron transporter 1, now referred to as *piuBCDA*) (Figure 1) (Brown et al., 2001a). A second locus also discovered in this study was termed *pit2ABCD* (now referred to as *piaABCD*). Recently, PiaA was crystallized, revealing the ability of this membrane protein to bind ferrichrome, a hydroxamate siderophore (Cheng et al., 2013). While previous data determined that the pneumococcus does not produce siderophores, this new PiaA data may suggest that *S. pneumoniae* is capable of stealing holo-siderophores produced by other bacteria in the human host. This could possibly act as an iron source, and has been previously reported to be an iron-acquistion mechanism in other pathogenic bacteria (Hibbing et al., 2010).

For both the *piu* and *pia* loci, a single operon encoded a putative lipoprotein receptor, a putative ATPase, and two transmembrane permease proteins. All mutants had impaired growth abilities in cation-free media, with the double *pia/piu* mutant having the largest defect. Growth defects were reversed by the addition of FeCl_3_ and FeCl_2_ but not by that of ferritin or lactoferrin. Also, Hb partially restored the growth of the individual mutants but not that of the double mutant. This partial rescue of growth in the presence of Hb may be due to the ability of Hb or hemin to be imported through the Piu/Pia transporters. Of note is that during the growth experiments, hemin may also be released from Hb via spontaneous dissociation, and the oxidized iron in hemin could be dissociated under these conditions (Hargrove et al., 1997; Tsuruga and Shikama, 1997). Therefore, the Piu/Pia transporters could target this fresh heme-iron pool for import.

To corroborate the finding that one or both iron transporters have a role in iron import, the iron-dependent antibiotic streptonigrin was used to determine bacterial sensitivity (Brown et al., 2001a). This bacteriocidal antibiotic requires free iron in the cytosol of bacteria; therefore, bacteria are resistant to this drug when their iron import systems are disrupted. The individual mutants both showed ~10-fold less sensitivity to streptonigrin than did the WT strain, suggesting that the mutants had less iron accumulate within the cytosol of the single/double mutants. The double mutant was even more resistant to the iron-dependent killing via streptonigrin. These data indirectly show that the loss of one or both iron import systems reduces iron import. This study also demonstrated direct iron uptake into the cytosol and indicated that significantly lower levels of iron accumulate within the cytosol (Brown et al., 2001a). These data clearly indicate the role of both the Piu and Pia systems in mediating iron uptake by the pneumococcus.

A third operon was discovered to contain genes encoding proteins similar to the already characterized iron import systems (Hoskins et al., 2001; Brown et al., 2002). The new putative iron import system was named *pitADBC* (Figure 1). The *pitA* gene encoded a lipoprotein iron receptor; *pitD* encoded an ATPase, and *pitB* and *pitC* encoded transmembrane permease proteins. At an amino acid level, PitA has more homology to *Streptococcus equi* and *Streptococcus pyogenes* PitA-homologs than to the pneumococcal PiuA and PiaA. The single *pitA*, *piuB*, and *piaA* mutants have WT-like growth characteristics in several growth media (Brown et al., 2002). However, growth is delayed in chelated media, and exogenous FeCl_3_ partially restores this growth defect. In contrast, adding Δ*pitA* to the double *piu/pia* mutant further reduces growth in all media tested, and this growth was partially restored in cation-free media by adding FeCl_3_ (Brown et al., 2002). These data indicate that some degree of specificity exists among these partially redundant systems.

### Regulation of iron transport

Although iron import systems are needed for the pneumococcus to acquire iron, this acquisition process must be tightly regulated at a transcriptional level for optimal detection of and response to the environmental stimuli encountered during infection. Because the pneumococcus inhabits multiple body sites, it is extremely important to detect extracellular and intracellular nutrient levels. Two-component systems (TCSs) can regulate such events, and studies to determine the role of TCSs within the pneumococcus revealed the existence of 13 such systems and one orphan response regulator, RitR (Lange et al., 1999; Throup et al., 2000; Tettelin et al., 2001). The results of studies initiated by Throup et al. and expanded upon by Ulijasz et al. show that the RitR (repressor of iron transport) regulator is required during murine lung infection (Throup et al., 2000; Ulijasz et al., 2004). Additional data show that this regulator may repress iron acquisition genes in high-iron environments.

Global transcriptional profiling efforts to determine the genes under control of RitR identified 54 genes, with 17 repressed and 37 activated in the pneumococcal WT R800 strain (Ulijasz et al., 2004). Notable changes in gene expression for this review were the following: (i) *piuB* and *piuA* (but not *piuC* or *piuD*), which showed the highest degree of differential gene expression and were repressed in WT R800; (ii) increased expression of Dpr homologs, which are iron-storage peroxide-resistance proteins, and the iron-binding alcohol dehydrogenase AdhE, both of which are proposed to protect bacterial cells against H_2_O_2_ damage (Echave et al., 2003; Pulliainen et al., 2003); (iii) HemH, which is responsible for the final step in heme synthesis (Panek and O'Brian, 2002); and (iv) MutY, an iron-sulfur protein that is required for A/G DNA mismatch repair, which occurs during oxidative stress (Michaels et al., 1992; Grollman and Moriya, 1993; Samrakandi and Pasta, 2000). Therefore, it seems that RitR is important for the controlled expression or repression of genes involved in oxidative stress response and protection.

To further analyze the effect that RitR has on the iron-uptake system Piu, streptonigrin sensitivity was measured to indirectly detect iron uptake (Ulijasz et al., 2004). When grown in iron-deplete media with no iron supplementation, the WT R800 and Δ*ritR* strains grew to similar levels and were resistant to streptonigrin. However, when Fe^2+^ or hemin was added to the media, only the Δ*ritR* mutant had increased susceptibility to streptonigrin killing (Ulijasz et al., 2004). These findings suggest that when RitR is absent, iron uptake is dysregulated and the metal hyper-accumulates in the cytosol. However, adding Fe^3+^ and Hb did not increase the susceptibility of either strain to streptonigrin, suggesting that the pneumococcus cannot use these iron sources under these conditions. These Hb results are in contrast to those previously discussed in this review (Tai et al., 1993). Additionally, the *ΔritR* strain is hyper-susceptible to H_2_O_2_ damage but not to the superoxide anion. The fact that RitR did not alter the expression of SodA is most likely responsible for maintenance of the resistance to oxidative stress caused by the superoxide anion. However, without a catalase, the pneumococcus would become increasingly susceptible to H_2_O_2_ damage, which would be further amplified by the higher intracellular Fe^2+^ concentrations that are present when RitR is absent.

Because RitR is a regulator of gene transcription that may be affected by differing Mn and Fe levels, Ong *et al*. analyzed gene expression of WT and Δ*ritR* strains grown in media with differing Fe:Mn ratios (Ong et al., 2013). The WT strain expressed two-fold more *ritR* in high-Fe media than in high-Mn media. This is consistent with reports that iron upregulates *ritR* (Ulijasz et al., 2004). The expression of *spxB*, which generates H_2_O_2_, was higher in the *ritR* mutant when grown in high-Fe media than when grown in high-Mn media. This finding supports the detection of increased H_2_O_2_ and oxidative stress in the mutant grown in high-Fe media (Ong et al., 2013). Also, because high Fe itself can produce oxidative stress, the researchers analyzed whether levels of NADPH, which can detoxify ROS, are changed in high-Fe environments. Additionally, *gnd*, which is involved in pneumococcal NADPH generation and is directly upstream of *ritR*, is expressed at a higher level in WT and *ritR* mutant cells grown in high-Fe media than it is in those grown in high-Mn media (Lanie et al., 2007; Ong et al., 2013). This expression pattern is further enhanced in the *ritR* mutant. Compared to the WT and *ritR*-complemented strains, the *ritR* mutant also expresses elevated levels of *zwf*, another gene essential for NADPH generation, when grown in high-Mn media. Additionally, the WT and complemented strains express 12-fold higher levels of the *psaA* gene in high-Fe media than in high-Mn media. However, the *ritR* mutant did not have increased *psaA* gene expression, suggesting that RitR may have a role in the expression of *psaA*.

Iron may also serve as a signal for a number of pneumococcal processes. Proteomic analysis of pneumococci cultured under iron-limiting conditions revealed altered expression of PsaA and numerous proteins involved in cellular stress responses and biofilm formation (Nanduri et al., 2008). The role of iron in biofilm formation has also been directly demonstrated, as supplementation of Fe^3+^ enhanced biofilm formation, and iron chelation severely impaired formation of these structures (Trappetti et al., 2011). The central regulator in the case of iron-dependent biofilm formation appears to be LuxS, which also controls expression of the iron importer PiuA (Trappetti et al., 2011). LuxS has been implicated in numerous cellular processes and is central for coordinating the formation of biofilms both on inert surfaces and on human respiratory cells (Vidal et al., 2011, 2013). These findings underscore the importance of iron in the regulation of diverse cellular processes in the pneumococcus.

### Role of iron transporters in pneumococcal pathogenesis

To determine the roles of Piu and Pia iron transporters in virulence, both pneumonia and a systemic infection were studied in murine models (Brown et al., 2001a). No differences were seen in survival between WT and individual-mutant strains. However, all mice infected with the double mutant survived the pneumonia infection. In contrast, the double mutant still caused 90% mortality during systemic infection, although the time-to-death was significantly delayed. Mixed infections were used to determine a competitive index between the WT strain and each mutant strain to better understand the roles of each iron import system in virulence. Here, the *pia* deletion was more attenuated in both the pneumonia and systemic infectious routes, suggesting that this iron importer is more important for pathogenesis. Also, the double mutant was extremely attenuated for virulence, with no bacteria recovered from the spleen 24 h after IP injection and few recovered from lungs after intranasal inoculation (Brown et al., 2001a). These data suggest that Piu and Pia transporters function independently yet synergistically and that, although each can compensate for the loss of the other, Pia may be more important for the viability of the pneumococcus during invasive infection.

These differences in virulence attenuation suggest that (1) the single *piu* or *pia* mutants are still virulent, most likely due to the function of the second import system; and (2) the iron requirements and availability in the lungs differ greatly from those in the bloodstream (Figure 1). It may be that the pneumococcus requires both iron transporters during the initial infection in the lungs for invasion and systemic infection to occur. However, the precise iron requirements and iron level availability during nasal colonization remain to be determined. Also, the possibility of a third iron-uptake transporter system, which would explain the low attenuation of virulence of the single mutants in this study, was highlighted in a 2002 paper from the same group (Brown et al., 2002).

In further support of the iron-uptake defect, the single mutations in all three iron importers caused similar sensitivity to streptonigrin, and each was more resistant than was the WT (Brown et al., 2002). Furthermore, the triple mutant was highly resistant to high doses of the drug, up to 20 μg/mL. Also, ^55^FeCl_3_-uptake was severely impaired in the triple mutant compared to the *piu/pia* mutant strain. The small amount of iron import in the triple mutant may be from another unknown cation importer non-selectively pumping in iron. Since the iron import was additive, it was not surprising that the deletion of *pit* did not further decrease virulence in a pneumonia mouse model of infection but did slightly impair the mutant's ability to cause systemic infection. Also, adding the *pit* mutation to the double *piu/pia* strain did not increase the virulence attenuation in either form of infection. Finally, *piaA* was shown to be transcribed at a higher rate than any *pit/piu* components, consistent with the view that this is the dominant iron import system (Brown et al., 2002). Together, these data show the relative contributions and redundancies of the three iron import systems during pneumococcal infection.

The role of RitR in the pathogenesis of the D39 strain was tested via systemic intraperitoneal infection (Ong et al., 2013). Although the mice infected with Δ*ritR* had slightly increased survival times, all mice eventually succumbed to infection. Furthermore, no differences were detected in the concentration of bacteria in the blood 24 h after infection with either the WT or mutant strain. This outcome was also seen in an intranasal challenge mimicking pneumonia. These data suggest that RitR is not needed for pneumococcal survival in systemic or pneumonia murine models of infection, contrasting those previously published (Ulijasz et al., 2004). The differences in the strains used (i.e., encapsulated D39 vs. non-encapsulated R800) as well as the infection model system may account for these contrasting virulence results.

As discussed, Ong et al. determined that RitR regulated iron import and manganese, which was needed for protection against oxidative stress that can occur when iron levels are high (Ong et al., 2013). To further understand the impact of RitR on virulence, the virulent encapsulated D39 strain and an isogenic Δ*ritR* mutant were analyzed for their growth in media with differing Fe:Mn ratios. The data suggested that RitR expression is essential for the growth of pneumococci in high-Fe:low-Mn media and that Mn^2+^ may provide a protective advantage when Fe^2+^ levels are in excess (Ong et al., 2013). Therefore, the authors hypothesized that the Mn-dependent SodA may mediate growth restoration when Mn^2+^ levels increase, protecting the bacteria against superoxides generated in the cytosol when the concentration of Fe^2+^ is high. A *sodA/ritR* double mutant was constructed and analyzed in growth experiments. In high-Fe:low-Mn media, exogenous Mn^2+^ still restored growth, suggesting that the Mn-mediated protection is not attributed to the oxidative protection of SodA. Regardless, these data suggest that Mn^2+^ can rescue the growth of the *ritR* mutant in high-Fe by reducing intracellular iron, and subsequently, oxidative stress.

### Targeting iron importers for vaccines

The pneumococcal ABC iron transporter systems elicit a strong antibody response during systemic infection, and these responses can protect against a pneumococcal infection (Brown et al., 2001b; Jomaa et al., 2005). This strategy has been used against many pathogens: in fact, iron import systems have previously been the target of a vaccine for *Staphylococcus aureus* (Ebert et al., 2010; Kim et al., 2010; Joshi et al., 2012; Pancari et al., 2012; Zapotoczna et al., 2013). Therefore, studies established by Brown et al. and expanded upon by Jomaa et al. used two of the iron import systems, Piu and Pia, as possible vaccine candidates (Brown et al., 2001b; Jomaa et al., 2005, 2006). Because PiuA and PiaA proteins are found in all pneumococcal serotypes tested, each protein was used (individually and together) to immunize mice for antibody responses and protection from subsequent pneumococcal challenge (Brown et al., 2001b). Specific antibody responses were detected against both proteins, with both PiuA and PiuA antibodies having cross-reactivity for each other. Although all of the mice immunized with adjuvant-only succumbed to the infection, both PiuA- and PiaA- immunized mice displayed improved survival, demonstrating that immunization with the iron import proteins increases survival rates and confers significant protection against systemic infection. As expected, immunization with both antigens further reduced the mortality rate. Passive immunization with antisera against PiaA-PiuA also significantly delayed time-to-death, indicating that immunization is at least partially antibody-mediated (Jomaa et al., 2005, 2006). Together, these data demonstrate that the lipoprotein components of two iron import systems can be used as a protein-based vaccine to protect against systemic pneumococcal infection.

In 2006, Whalan et al. determined that the *piaA* gene was 100% conserved in all typical pneumococci tested, which included 27 different serotypes, but was not found in any oral streptococci, including the closely related *Streptococcus mitis* (Whalan et al., 2006). However, *piuA*, was found in only 20 pneumococci serotypes, with a very low level of nucleotide divergence (0.3%), and was also found in *S. mitis* and *S. oralis*, with a higher level of divergence (10%). Regardless, these data further support the idea that PiuA and PiaA could be viable protein-vaccine candidates.

Evidence emerged in 2005 that PiuA and PiaA elicit a strong immune response in humans: patients with laboratory-confirmed pneumococcal septicemia had elevated levels of antibodies directed against these two antigens in the convalescent phase of the infection but not in the acute phase (Whalan et al., 2005). This finding suggests that humans are exposed to PiuA and PiaA during systemic infection and mount antibody responses against these antigens, as occurs in infected mice. It also suggests that these two iron import proteins have the potential to be included in a protein-based vaccine. In the patient sera tested, eight different pneumococcal serotypes had specific antibody responses against PiuA and PiaA, demonstrating serotype-independent cross-reactivity. Also, both lipoproteins were immunogenic in healthy 7-month-old infants, suggesting that immunogenicity can be quickly established after birth, most likely through normal pneumococcal colonization of the nasopharynx (Whalan et al., 2005). These experimental and clinical observations indicate that targeting iron import may prove to be an effective vaccine strategy against the pneumococcus.

## Copper

Copper is toxic to a multitude of microorganisms, including bacteria, fungi, and viruses. Accordingly, operating rooms have begun using copper surfaces and surgical tools to reduce the risk of nosocomial infections (Salgado et al., 2013). To resist copper toxicity, bacteria have developed highly efficient copper export systems, many of which have important roles during infection as recently reviewed (Samanovic et al., 2012).

### The cupA copper efflux system

*S. pneumoniae* has a highly conserved *cop* operon containing three genes: *copY*, the operon regulator; *cupA*, a copper transport protein with homology to a cupredoxin; and *copA*, the P-type ATPase copper exporter (Figure 1) (Shafeeq et al., 2011b). Several additional proteins have been identified as putative copper homeostasis proteins in *S. pneumoniae* (CutC, CtpC, and CtpE). Although these putative copper homeostasis proteins are upregulated during infection, they are not upregulated during copper stress, putting their precise function in *S. pneumoniae* into question (Shafeeq et al., 2011b). One possibility is that the relative abundance of other trace metals during infection is distinct to the *in vitro* media conditions used, resulting in increased expression. The precise function of these other proteins in copper homeostasis and pathogenesis remains unknown. It should also be noted that the mechanism of copper import in the pneumococcus has yet to be elucidated.

### Regulatory control of copper efflux

Transcriptional control of the *cop* operon in pneumococci is under the tight regulatory control of CopY, which represses the *cop* operon by binding the promoter region of the operon (Shafeeq et al., 2011b). Data obtained from other organisms show that once CopY binds copper, its affinity for DNA is greatly decreased, thus allowing transcription of the operon (Portmann et al., 2006). One atom of a metal inside the bacterial cell corresponds to ~1.5 nM concentration, assuming a bacterial volume of ~10^−15^ liters per cell. CopY's affinity for copper has been estimated to be in the zeptomolar range (10^−21^) in other bacterial species, equating to less than one molecule of copper per bacterium and supporting the case that free intracellular copper is extremely detrimental to bacterial (Changela et al., 2003). Additionally, in other bacterial species, CopY controls expression of lactate oxidase, which can function to scavenge molecular oxygen (Barre et al., 2007). LctO is also involved in hydrogen peroxide production in streptococci (Kietzman and Caparon, 2010). It is unknown whether the pneumococcal CopY controls *lctO* expression or plays a role with SpxB, which is encoded immediately downstream of the *cop* operon.

### Molecular mechanism of copper efflux

Recent structural data indicate that copper export is mediated by CupA transporting copper from the protein's low-affinity site to its high-affinity site (Shafeeq et al., 2011b; Fu et al., 2013). This system provides an extremely elegant model for the efficient trafficking of intracellular copper for efficient efflux because both CupA and CopA are membrane-anchored proteins (Fu et al., 2013). It remains unclear whether CupA and CopY interact to transfer the copper and precisely how the membrane-bound CupA acquires free copper inside the bacteria. The results of homology studies suggest that CupA has cupredoxin activity, reducing soluble Cu^2+^ to insoluble Cu^1+^, but direct evidence has not been shown (Fu et al., 2013). Pneumococcus can produce millimolar amounts of hydrogen peroxide, which reacts with Cu^1+^ to form water and free radicals via Fenton chemistry (Pericone et al., 2003). In theory, free-radical production could be circumvented by CupA sequestering Cu^1+^ after reduction and directly transferring it to CopA for export from the bacteria or by active uptake of glutathione, which converts hydrogen peroxide to water, thus relieving the stress of the free-radical formation by removing both precursors (Potter et al., 2012). The observation that glutathione uptake deficient pneumococci are more susceptible to copper stress supports this notion (Potter et al., 2012).

One of the major underlying questions about intracellular copper accumulation is the precise mechanism of toxicity. Recent data suggest that copper alone is not sufficient to cause oxidative damage in bacteria; therefore, it is not unreasonable that copper's toxic effects are mediated through another pathway (Macomber et al., 2007). Copper is very stable as represented in the Irving-Williams series, so it could displace metals in crucial bacterial enzymes, leading to their inactivity (Milicevic et al., 2011). Accordingly, copper negatively affects iron-sulfur clusters of dehydratases, inhibits branched chain amino acid biosynthesis, photosystem oxidase, and displaces manganese in SOD as additional mechanisms of toxicity (Batinic-Haberle et al., 1997; Macomber and Imlay, 2009; Azzouzi et al., 2013). Ribonucleotide reductase protein, NrdF, and coupled factor NrdE are both necessary for the aerobic nucleotide synthesis pathway in pneumococci and may interact with copper, as may other proteins involved in aerobic nucleotide synthesis, such as NrdI (Sun et al., 2011). Thus, it is also possible that copper is causing toxicity by inhibiting nucleotide synthesis or other essential metabolic processes.

### Copper and pathogenesis

Proteins involved in copper homeostasis have been implicated at multiple stages of pneumococcal pathogenesis. The inability of the pneumococcus to functionally export copper is detrimental to the cell, with Δ*copA* mutants being more susceptible to copper toxicity than are wild-type bacteria (Shafeeq et al., 2011b; Fu et al., 2013). Mice intranasally infected with Δ*copA* mutants have an increased survival rate, likely due to the mutant's inability to grow in the host's nasopharynx (Shafeeq et al., 2011b). The results of signature-tagged mutagenesis screening and transposon sequencing implicate CopA involvement in pneumococcal fitness in both the nasopharynx and the lung (Hava and Camilli, 2002; van Opijnen and Camilli, 2012). Virulence data for the Δ*copY* and Δ*cupA* mutants are unavailable, however, transposon sequencing results indicate that no significant defects in either lung infection or nasopharyngeal colonization would occur when these genes are disrupted (van Opijnen and Camilli, 2012). This finding is in agreement with previous findings that Δ*copY* mutants have wild-type tolerance of copper, Δ*cupA* mutants have a similar tolerance to that of Δ*copA* mutants depending on the media, and Δ*cupA* and Δ*copY* mutations have little or no effect on virulence, respectively (Shafeeq et al., 2011b; Fu et al., 2013).

The attenuation of the Δ*copA* mutant during infection may be partially explained by the activity of the ATP7a copper transporter expressed in macrophages and other cells because this strain is unable to export copper. ATP7a has been implicated in the elimination of pathogens via importing excess copper into the phagolysosome to mediate more-effective bacterial killing (White et al., 2009). Deletion of ATP7a in murine models resulted in severe growth defects and premature death, making investigation into susceptibility to infection difficult (Wang et al., 2012b). Advances into the role of ATP7a will be facilitated by the recent cell line–specific conditional knockouts of this transporter in murine systems (Wang et al., 2012a,b). Such tools will allow for refined investigations into the role of this copper transporter during the progression of infection. Another major question that remains unanswered is why the pneumococcus uptakes copper at all. Copper-dependent enzymes have not been found, yet the amount of intracellular copper and the universal conservation of dedicated export machinery indicate that a copper acquisition system may exist. Future studies of the precise molecular interactions that facilitate efficient copper export and of the precise role of *cop* operons and other predicted copper homeostasis pathways will provide insight into the essential copper homeostasis pathways.

## Zinc

In contrast to copper's toxicity in *S. pneumoniae*, zinc has both beneficial and detrimental effects in bacteria, with several classes of bacterial proteins, such as metalloproteases and transcriptional regulators, relying on zinc for function although high concentrations of zinc are toxic (Guerra et al., 2011; McDevitt et al., 2011; Milicevic et al., 2011; Bek-Thomsen et al., 2012; Menon and Govindarajan, 2013). In a recent review, Shafeeq et al. comprehensively outline the literature on zinc's homeostasis, transport, and involvement in the pathogenesis of streptococci (Shafeeq et al., 2013). During infection, the phagolysosomes of macrophages either sequester zinc, reducing the bacteria's ability to use zinc for essential cellular processes, or overwhelm bacteria with zinc, leading to toxicity (Kehl-Fie and Skaar, 2010; Botella et al., 2011). Switching between these innate immune responses may be due to the perceived level of toxicity of the bacteria, however, further investigation would be needed to understand the exact mechanisms (Botella et al., 2011). To account for both situations inside the host, *S. pneumoniae* have zinc influx and efflux pumps.

### Zinc acquisition

*S. pneumoniae* encodes five proteins directly involved in zinc acquisition: zinc importers AdcA and AdcAII, permease AdcB, ATPase AdcC, and transcription regulator AdcR (Figure 1) (Dintilhac and Claverys, 1997; Dintilhac et al., 1997; Bayle et al., 2011). The transporters AdcA and AdcAII have overlapping specificity for zinc *in vitro*, and the combined Δ*adcA*/Δ*adcAII* mutant is deficient in zinc uptake. Furthermore, strains having deletions of both genes have deficiencies in growth in low-zinc environments and have severe colonization defects in intranasal and intraperitoneal infection models (Bayle et al., 2011). Deletion of the permease AdcB also confers deficiency in growth in low-zinc environments, with a slight defect in growth under normal conditions as well.

The Pht family of proteins (PhtA, PhtB, PhtD, and PhtE) may also be involved in zinc homeostasis. These proteins are upregulated in the presence of excess zinc but are under the control of AdcR (Ogunniyi et al., 2009; Shafeeq et al., 2011a). The single Δ*phtD* mutation does not affect zinc uptake or growth, but the mutation of all four Pht proteins increases growth in the presence of excess zinc, leading to the suggestion that these proteins may be zinc scavengers or involved in zinc uptake. However, the results of direct binding assays involving these proteins and zinc have not been published (Bayle et al., 2011; Rioux et al., 2011).

### Zinc efflux

In levels of zinc that are higher than equilibrium, *S. pneumoniae* mediates resistance to zinc toxicity by upregulating proteins such as AdhC, a glutathione-dependent alcohol dehydrogenase, and CzcD, a zinc export system protein in the cation diffusion facilitator family, both of which defend against the reactive nitrogen byproducts of zinc (Kidd et al., 2007; Kloosterman et al., 2007; Kimaro Mlacha et al., 2013; Shafeeq et al., 2013). Gene transcript levels of *czcD* and *adhC* are elevated in more-invasive strains of pneumococcus, such as TIGR4, and although initial reports showed that AdhC was essential for lung infection, it has since been shown that neither *czcD* nor *adhC* contribute to overall pathogenesis (Hava and Camilli, 2002; van Opijnen and Camilli, 2012; Kimaro Mlacha et al., 2013). However, just as it does with zinc import, *S. pneumoniae* may have overlapping functionality in zinc efflux, in which case single mutations may not yield discernible phenotypes under general conditions.

## Other transition metals

Although little is known about the role of cobalt, nickel, and other transition metals not already mentioned in this review, it is likely that these metals can also enter the bacterium, so *S. pneumoniae* likely possesses the ability to export these metals. Several uncharacterized putative E1-E2 P-type ATPases exist in the *S. pneumoniae* genome that could facilitate import or export these additional transition metals. The *czc* locus has been shown to mediate cobalt and cadmium resistance in other organisms, however, there is no direct evidence of such activity in *S. pneumoniae* (Nies, 1992). Nevertheless, *czcD* transcript levels are upregulated in the presence of both cobalt and nickel, implying that CzcD could indeed be exporting these metals. Additionally, Sun *et al*. have described a group of proteins that likely bind cobalt and nickel, with the cobalt-binding proteins found in ribosomal structures and having RNA binding and structural activity and the nickel-binding proteins having ligase activity (Sun et al., 2013). However, the roles of these transporters in pneumococcal metabolism and virulence have not been determined. Cobalamin (vitamin B12) is a cobalt-binding compound that some intestinal bacteria use, however, no evidence exists that *S. pneumoniae* uses vitamin B12 (Giannella et al., 1971). It should also be noted that the pneumococci encode numerous additional ABC transporters and putative efflux systems, although the substrates of these systems and their roles during infection remain unknown.

## Conclusions

The environments encountered by the pneumococcus in the human body during both colonization and infection vary greatly in terms of the bioavailability of trace metals used as co-factors by both host and pathogen. As such, metal homeostasis is vital not only to fight bacterial infections but also to survival of the pathogen. Building upon our understanding of the metal import and export machinery of *S. pneumoniae* not only improves basic science knowledge of metal trafficking and usage in the bacteria but also offers additional therapeutic targets for inhibiting bacterial infections. Because many of the metal acquisition systems of the pneumococcus are both surface-exposed and important for invasive infection, they remain attractive vaccine candidates for protein-based vaccines. Another potential avenue for future therapeutics would be compounds that specifically target these essential bacterial uptake and efflux systems. The feasibility of this approach was recently demonstrated in *Staphylococcus aureus*, whereby using small molecules to activate the regulatory system controlling heme uptake perturbed the central metabolism and was toxic to the bacterium (Mike et al., 2013). Extension of such screens to other essential metal homeostasis systems may provide a new avenue for novel therapeutics to target bacterial pathogens.

### Conflict of interest statement

The authors declare that the research was conducted in the absence of any commercial or financial relationships that could be construed as a potential conflict of interest.
